# Real world clinical outcomes of treatment of cannabis-induced psychosis and prevalence of cannabis-related primary psychosis: a retrospective study

**DOI:** 10.1186/s12888-024-06075-6

**Published:** 2024-09-27

**Authors:** Onrumpha Chuenchom, Thanarat Suansanae, Lumsum Lukanapichonchut, Somporn Suwanmajo, Chuthamanee Suthisisang

**Affiliations:** 1 Department of Pharmacy, Faculty of Pharmacy, Mahidol University, Bangkok, 10400 Thailand; 2 Department of Medicine, Princess Mother National Institute on Drug Abuse Treatment, Pathum Thani, 12130 Thailand; 3 Department of Pharmacy, Princess Mother National Institute on Drug Abuse Treatment, Pathum Thani, 12130 Thailand; 4 Department of Pharmacology, Faculty of Pharmacy, Mahidol University, Bangkok, 10400 Thailand; 5 ASEAN Institute for Health Development, Mahidol University, Nakhon Pathom, 73170 Thailand

**Keywords:** Cannabis, Cannabis-induced psychosis, Treatment of cannabis-induced psychosis, Cannabis-related primary psychosis

## Abstract

**Background:**

Current treatment of cannabis-induced psychosis (CIP) focus on the presenting symptoms of individual patient. Therefore, the objective of this study was to investigate the efficacy of pharmacological treatment for CIP in a retrospective manner.

**Methods:**

A retrospective chart review study was conducted at the Princess Mother National Institute on Drug Abuse Treatment (PMNIDAT), Thailand. Patients aged more than 12 years who met the International Classification of Disease-10 (ICD-10) criteria of CIP, had recorded of cannabis use in medical chart, and had positive urine test of cannabis on the first day of admission from October 2013 to September 2019 were enrolled. The primary outcome was the efficacy of pharmacological treatment of CIP. Brief Psychotic Rating Scale (BPRS) on the first day and weekly after receiving treatment were used to assess the primary outcome.

**Results:**

Four hundred and three medical charts with diagnosis of CIP were enrolled into the study and only 317 charts were analyzed. Most of them were male with an average aged of 21.0 (19.0–24.0) years old. All of them used smoked cannabis from dried leaves and flowers of cannabis plant. The presented symptoms on admission were psychosis, mood symptoms, sleep problems, weight loss, and cognitive problems (100%, 64%, 61%, 11%, and 7%, respectively). Baseline BPRS score of the first day of admission was 55.2 ± 9.6. Majority of patients received antipsychotic (98.7%) followed by the combination of antipsychotics with benzodiazepines (34.5.%), antipsychotics with antidepressants (14.4%) and antipsychotics treatment with antidepressants and benzodiazepines (25.9%). Only few patients received antipsychotic monotherapy (17.9%). Risperidone was the most frequently prescribed antipsychotics (83.6%). Mean equivalence dose of risperidone was 8.0 ± 5.9 mg/day. The average hospital length of stay was 28 days (range 22-31). BPRS at 22 days significantly improved compared to the first day of admission (*p* < 0.001). Schizophrenia was diagnosed in 7% at 1.3 years of follow up.

**Conclusion:**

Antipsychotics was still a key psychotropic drug for treatment of CIP. The symptoms were decreased rapidly and sustained among the treatment period. However, antidepressants and benzodiazepines were commonly used for treatment of other symptoms beyond psychosis.

**Trial registration:**

ClinicalTrials.gov ID: NCT04945031 (Registration Date: 30 June, 2021).

## Introduction

Cannabis is a widely used psychoactive substance around the world, and the problem of cannabis misuse is still a big concern. There are many reports of cannabis users experiencing psychotic symptoms due to a tetrahydrocannabinol (THC)-induced increase in dopaminergic activity in the mesolimbic dopaminergic system through coupling with cannabinoid receptor 1 (CB_1_) receptors [1]. Moreover, chronic cannabis usage may increase the risk of developing schizophrenia, bipolar disorder or cognitive impairment [2–4].

From 2010 to 2015, a European study comparing 901 patients with their first episode of psychosis to 1,237 patients in the control group, it was found that patients who used cannabis were 3.2 times more likely to have psychosis symptoms than patients who did not use cannabis, and that the risk of psychosis increased in patients with a high THC level (THC greater than 10%) [5]. Psychosis from cannabis remains an important issue for the healthcare system in Thailand. Since 2018, Thailand has become the first Southeast Asian country to withdraw cannabis from the Narcotic Drugs List, and since then, thousands of cannabis shops and businesses have sprung up, especially in Bangkok and tourist spots. As a result, the number of people who do not use cannabis for medical purposes has been increasing due to its easy accessibility. We found that cannabis was the third most commonly used substance after amphetamine and alcohol (66.22%, 20.47%, and 5.03%, respectively) among patients admitted to the excellent center for treatment of substance use disorder in Thailand known as Princess Mother National Institute on Drug Abuse Treatment (PMNIDAT) in 2020 [6].

The current treatment aims to reduce target symptoms associated with cannabis use, especially psychosis. Antipsychotics with potent dopamine-2 (D_2_) receptor antagonistic effects are the main treatment of choice because they can block the D_2_ receptor at the mesolimbic pathway and therefore reduce psychotic symptoms from THC. The results from double-blind randomized controlled trials in CIP demonstrated that haloperidol, olanzapine, and risperidone were all effective, and there were no significant differences between the drugs for a 4-week duration [7, 8]. However, there were limited clinical studies of antipsychotics in cannabis-induced psychosis (CIP). Therefore, the purpose of this study was to investigate the efficacy of pharmacological treatment, especially antipsychotics, for CIP in real-world practice.

## Methods

### Subjects

A retrospective chart review was conducted at the PMNIDAT. The authors assert that all procedures contributing to this work comply with the ethical standards of the relevant national and institutional committees on human experimentation and with the Helsinki Declaration of 1975, as revised in 2008. All procedures involving human subjects/patients were approved by Institutional Review Board of Faculty of Dentistry/Faculty of Pharmacy, Mahidol University (MU-DT/PY-IRB 2019/074.3110; Oct 31, 2019) and the PMNIDAT, Department of Mental Health, Ministry of Public Health, Thailand (No. 010/2563; Nov 8, 2019). Informed consent for all patients, including those < 18 years old, was waived by the ethics committee of Institutional Review Board of Faculty of Dentistry/Faculty of Pharmacy, Mahidol University and the PMNIDAT. In addition, individual data had been collected by using a code that prevented browsing to an individual.

Data collection has been done after receiving IRB approval. All patients who were diagnosed with CIP according to International Classification of Disease-10 (ICD-10; version 2016, code F12.5) and were admitted at the PMNIDAT between October 2013 and September 2019 were enrolled in the study.

The inclusion criteria were: aged ≥ 12years old, had a history of cannabis use in their medical charts, and had a positive urine screening test for cannabis on the first day of admission. Exclusion criteria were psychosis from organic psychosis, unavailable essential data in medical records, and receiving medical treatment less than 7 days after admission.

All prescribed medications for treatment of CIP during admission were recorded. They were divided into two phases: acute phase (treatment on the first day of admission) and the maintenance phase (treatment when the patients were clinically stable such as before discharge from the hospital or during a rehabilitation program). The Brief Psychiatric Rating Scale (BPRS, 18 items) score was recorded on the first day and every 7 days after the first day of admission. BPRS scores showed the severity of psychiatric symptoms, on a scale from 1 to 7. Higher scores indicate more severity [9, 10]. A change in the BPRS score after receiving pharmacological treatment at discharge compared to the first day of admission was recorded.

In addition, all patients were monitored after their discharge from the hospital within the study period about the development of schizophrenia by the ICD-10 for schizophrenia (version 2016, code F20.0).

### Statistical analysis

All data were analyzed using the Statistical Package for the Social Sciences (SPSS) version 25. For this study, type I error was set at α = 0.05 and the power of the test was set at 80% (β = 0.2). A p-value < 0.05 was considered statistical significance. Demographic data and characteristics were reported as mean and standard deviation, or medians and interquartile. Gender, marital status, religion, location, medical welfare, education level, occupation, underlying disease, and drug allergy were presented as frequencies and percentages. The normality of each variable was assessed using the Kolmogorov-Smirnov.

The dose of psychotropic drugs was reported as mean and standard deviation, or medians and interquartile, as an equivalence dose of risperidone. The number of psychotropic drugs is presented as frequency and percentages.

The BPRS scores of days 8, 15, and 22 were compared to the first day by a paired t-test. An intention-to-treat (ITT) analysis was used in the analysis. In addition, factors associated with the changes in BPRS scores at day 1 and day 22 were tested by linear regression.

## Results

Four hundred and three medical charts with a diagnosis of CIP were screened, and only 317 charts with urine positive for cannabis were recruited for analysis. The 86 medical charts were excluded due to a negative urine test for THC.

The average age was 21.0 (19.0–24.0) year old, most of them were male (97.2%), single (86.7%), unemployed (56.2%), and had universal health coverage (83.9%). More than half of patients (64.3%) graduated from secondary or high school levels. Most patients (86.1%) had no underlying diseases. Their baseline characteristics were summarized (Table 1).

**Table 1:** Baseline characteristics

Characteristics	Value (*N* = 317)
Age, year [median (IQR)]	21.0 (19.0–24.0)
Gender, n (%)	
- Male	309 (97.2)
BMI (kg/m^2^), [median (IQR)]	20.1 (18.0- 22.9)
Marital status, n (%)	
- Single	275 (86.8)
- Married/ Couple	29 (9.1)
- Divorced/ Separate	13 (4.1)
Religion, n (%)	
- Buddhism	307 (96.8)
- Islam	64 (1.3)
- Christianity	6 (1.9)
Medical welfare, n (%)	
- Universal Health Coverage	266 (83.9)
- Social Security Scheme	4 (1.3)
- Self-pay	47 (14.8)
Education, n (%)	
- Elementary school	59 (18.6)
- Secondary school	204 (64.3)
- Diploma/ bachelor’s degrees	44 (13.9)
- Missing data	10 (3.2)
No underlying disease, n (%)	273 (86.1)
No Drug allergy	311 (98.1)
Occupation	
- Unemployed	178 (56.2)
- Student	67 (21.1)
- Employee	54 (17.0)
- Government official	4 (1.3)
- Others	12 (3.8)
- Missing data	2 (0.6)

The mean age of the first-time users of cannabis was 16.3 ± 3.4 years old, and the lowest age was 9 years old. All of them did not use cannabis for medical purposes, and they smoked cannabis from dried leaves and flowers of cannabis plants, with a median duration of use of about 5 years (range 3–7 years). The data on the history of drug use before admission was obtained from patient interviews recorded in medical charts. Approximately 38% of patients reported using cannabis about 4 holes per day before admission. Some patients used cannabis combined with other substances such as methamphetamine, alcohol, kratom, glue, heroine, and methadone (Table 2). About 90% of patients smoked cigarette in average half pack a day.

**Table 2:** History of substances used before admission

Drugs	Route of admission	Duration (year) [median (IQR)]	Daily dose of drugs per day [median (IQR)]	Last used (day) [median (IQR)]	Last dose of drugs per day [median (IQR)]
Cannabis (*n* = 317)	Smoked	5.0 (3.0–7.0)	5.0 (3.0–10.0) holes (*n* = 115) 1.0 (1.0–1.0) packs (*n* = 77) 3.0 (2.0–4.0) rolls (*n* = 51) 2.0 (1.0–3.0) cutting board (*n* = 31) 1.0 (1.0- 2.5) bongs (*n* = 18) 4.0 (3.0–25.0) times (*n* = 13) 100.0 (100–500) gram (*n* = 7) 1.0 (1.0–1.0) chunk (*n* = 5)	2 (1–5)	4.0 (2.0-9.3) holes (*n* = 122) 1.0 (1.0–1.0) packs (*n* = 64) 2.0 (1.0-3.5) rolls (*n* = 53) 1.0 (1.0–3.0) cutting board (*n* = 29) 2.0 (1.0–2.0) bongs (*n* = 19) 3.0 (1.0–5.0) times (*n* = 20) 400.0 (75.0-500.0) gram (*n* = 5) 1.0 (1.0–1.0) chunk (*n* = 5)
Cigarette (*n* = 288)	Smoked	5.0 (4.0–8.0)	12.0 (5.0–20.0) cigarettes	0*	10.0 (5.0–20.0) cigarettes
Amphetamine (*n* = 44)	Smoked (*n* = 42) Ingestion (*n* = 1) Injection (*n* = 1)	4.0 (1.0–9.0)	2.0 (1.0–5.0) tablets	10 (2.0–30.0)	2.0 (1.0–3.0) tablets
Alcohol (*n* = 42)	Ingestion	10 (5.8–13.5) (*n* = 6)	49.5 (25.8-126.4) grams (*n* = 10)	1.0 (0.75–3.25)	49.5 (25.8-221.2) grams (*n* = 10)
Crystal methamphetamine (*n* = 20)	Inhalation	2.5 (0.0-5.3)	0.5 (0.3-1.0) gram(*n* = 7) 1.0 (1.0-1.75) tak (*n* = 12) 5.0 (5.0–5.0) times (*n* = 1)	4 (1.0–7.0)	1.0 (0.0–1.0) gram(*n* = 7) 1.0 (1.0-1.3) tak (*n* = 10) 5.0 (5.0–5.0) times (*n* = 1)
Kratom (*n* = 19)	Ingestion	3.0 (1.0–6.0)	1.0 (1.0–2.0) liter (*n* = 7) 3.0 (1.5–4.8) glass (*n* = 4) 25.0 (7.3–30.0) leaves (*n* = 4) 2.0 (1.0) pot (*n* = 3) 1.0 (1.0–1.0) pack (*n* = 1)	2.0 (1.0–14.0)	1.0 (1.0–2.0) liter (*n* = 7) 3.0 (1.3–4.8) glass (*n* = 4) 25.0 (6.5–30.0) leaves (*n* = 4) 1.5 (1.0-2.8) pot (*n* = 4)
Alprazolam (*n* = 5)	Ingestion (*n* = 3) Inhalation (*n* = 2)	1.0 (0.35-3.0)	8.5 (4.0–10.0) tablets	15.0 (5.3–22.5)	5.0 (3.0–5.0) tablets
Glue (*n* = 4)	Inhalation	3.0 (0.3–6.5)	1 (1.0-3.3) cans of glue	34.5 (6.0–60.0)	1.0 (1.0–1.0) cans of glue
Heroine (*n* = 2)	Inhalation	1.0 (0.0)	1.0 (1.0–1.0)	9.5 (4)	1.0 (1.0–1.0)
Methadone (*n* = 1)	Ingestion	14 (14–14)	20.0 (20.0–20.0) ml	11.0 (11.0–11.0)	10.0 (10.0–10.0) ml

* Last used of this substance on the admission date

**Fig. 1:**
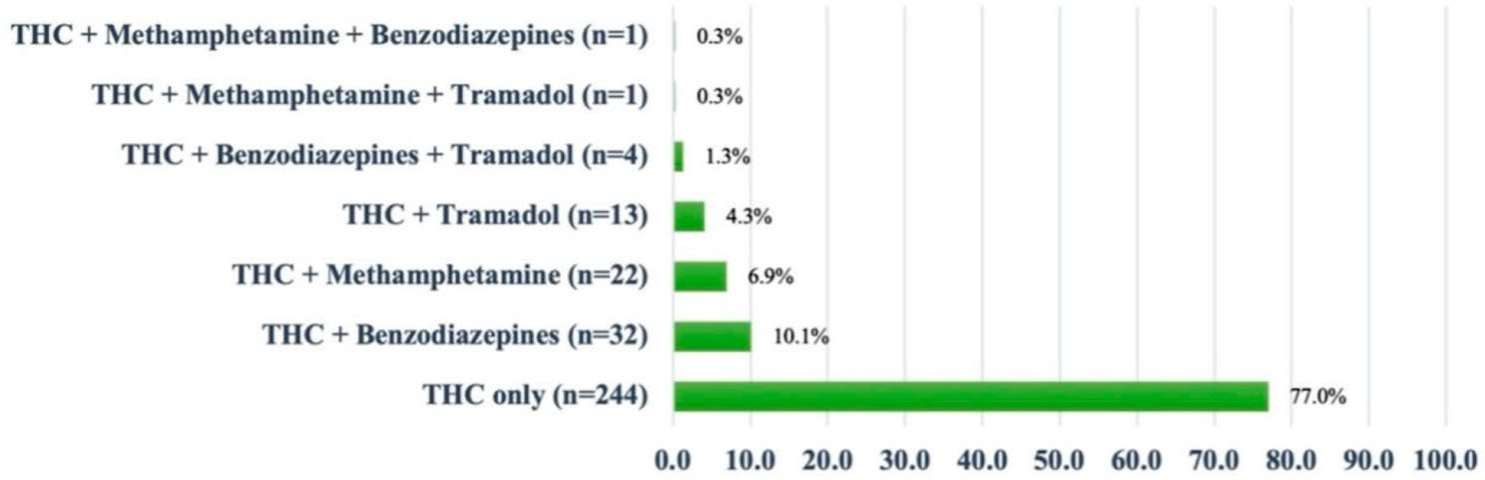
Urine screening test results for cannabis and other substances

When focusing on the urine screening test of substances on the first day of admission, we found that all patients had positive THC urine tests, and some of them had urine tests positive for methamphetamine, benzodiazepine, and tramadol (Fig. 1). THC level in the urine were performed in patients who had severe psychosis (*N* = 122/317; 38.5%), with a mean level of 110.5 (97.0-127.0) ng/mL.

The common chief complaints for admissions were hallucination (84.9%), delusion (82.6%), sleep insufficiency (61.2%), irritability (57.1%), and aggression (42.0%). These symptoms developed after receiving the last dose of cannabis for about 2 days (range 1–5 days).

In the acute phase of treatment (treatment on the first day of admission), nearly all patients received antipsychotics. About 1.3% of the admitted patients did not receive antipsychotics because there was no psychosis as a predominant symptom. More than half of them received antipsychotics and benzodiazepines, with or without antidepressants (Table 3). A combination of intramuscular (IM) haloperidol and intravenous (IV) diazepam was commonly used to control the psychotic symptoms. Oral antipsychotics that had been frequently prescribed were risperidone (83.6%), haloperidol (19.9%), and perphenazine (7.9%).

**Table 3:** Comparison of pharmacological treatment patterns between acute and maintenance phases for the treatment of CIP (*N* = 317)

Prescription patterns	Acute phase (*N*)	Maintenance phase (*N*)
Monotherapy with antipsychotics	56 (17.9%)	103 (32.9%)
Combination of antipsychotics with other psychotropic drugs		
• + Benzodiazepines	108 (34.5%)	5 (1.6%)
• + Antidepressants	45 (14.4%)	155 (49.5%)
• + Antidepressants and benzodiazepines	81 (25.9%)	7 (2.2%)
• Other combinations	23 (7.3%)	43 (13.7%)

Regarding maintenance phase treatment (treatment when the patients were clinically stable, such as before discharge from the hospital or during a rehabilitation program), antipsychotics remained a mainstay treatment. Risperidone (85.5%; median dose 2–4 mg/day) was the most prescribed antipsychotics, followed by haloperidol (47.3%; median dose 5–20 mg/day) and clozapine (14.8%; median dose 100–150 mg/day). Clozapine has been prescribed only in combination with other antipsychotics to target other symptoms beyond psychosis. Furthermore, there were few prescriptions of long-acting injectable antipsychotics in both acute (17/313, 5.4%) and maintenance phase treatment (45/313, 14.4%). Most of them were haloperidol decanoate (9.8%; median dose 100 mg per month [range 50–100]). The mean dose of antipsychotic treatment expressed as risperidone equivalence doses on day 1, day 8, day 15, and day 22 was 12.2 ± 6.9, 8.3 ± 5.3, 8.4 ± 5.9 and 8.0 ± 5.9 mg per day, respectively.

There was a concurrent use of antidepressants and benzodiazepines with antipsychotic medications in this study (Table 3). Few of them received more than two psychotropic drugs. During both the acute and maintenance phases of treatment, the most commonly prescribed antidepressant was sertraline, accounting for approximately 36.6% of prescriptions. The median dose of sertraline was 50 mg per day. Following sertraline, fluoxetine was the next most commonly prescribed antidepressant (18.3%). The median dose of fluoxetine was 20 mg per day. Reboxetine, although less frequently prescribed, accounted for approximately 2% of prescriptions with a median dose range of 4–6 mg per day. Additionally, benzodiazepines were prescribed more frequently during the acute phase than during the maintenance phase of treatment. Clonazepam was the most frequent prescribed (range 0.5-2 mg/day).

**Fig. 2:**
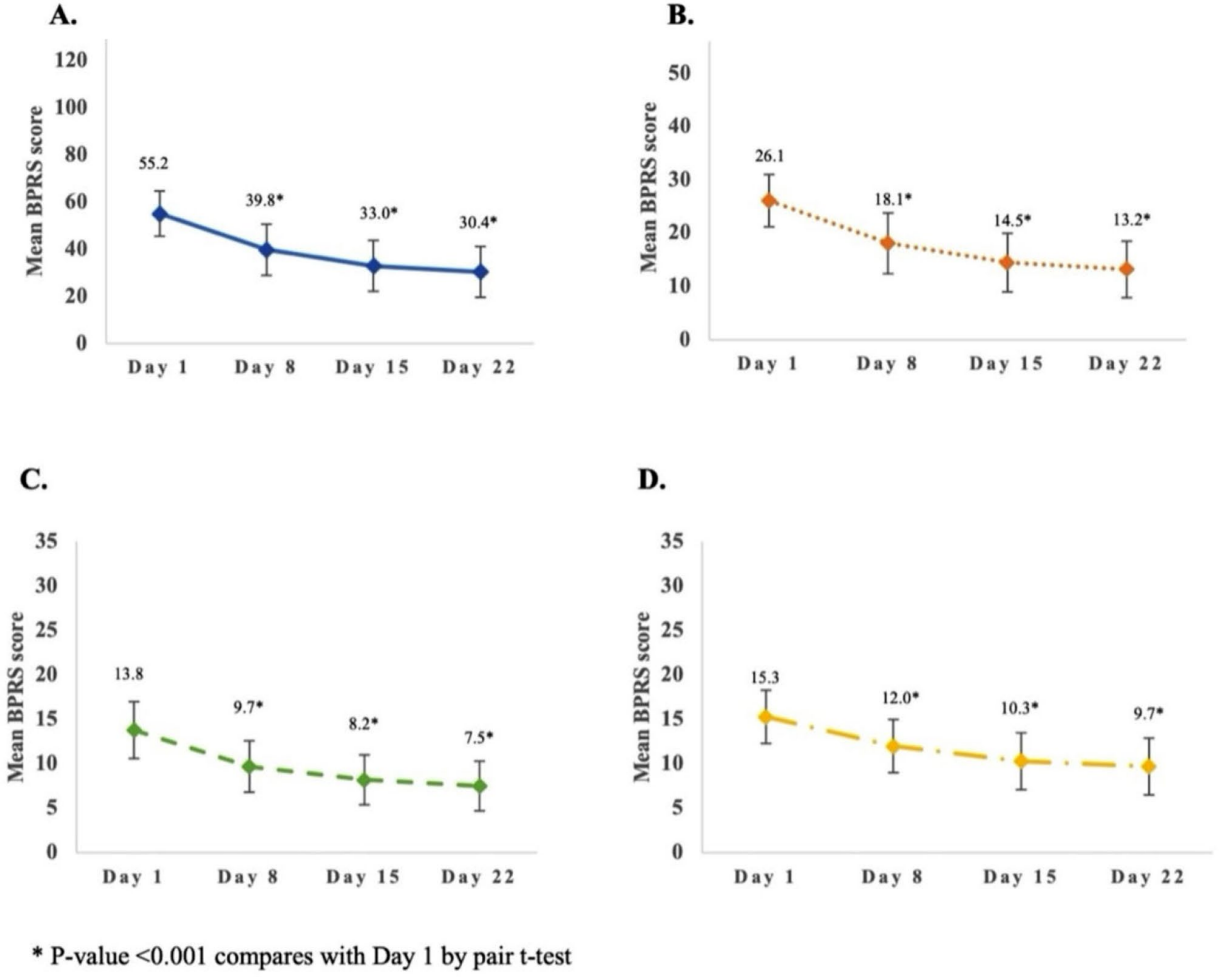
Changes of total BPRS score (**A**), positive scale (**B**), negative scale (**C**), and general scale (**D**) after receiving psychotropic drugs during the study period (*n* = 133)

On the first day of admission, the mean total BPRS score, positive scale, negative scale, and general scale were 55.2 ± 9.6, 26.1 ± 4.9, 13.8 ± 3.2, and 15.3 ± 3.2, respectively. The highest scores of each item on the positive, negative, and general scales were suspiciousness, uncooperativeness, and tension, respectively. The median length of hospitalization was 28 days (range 22–31 days). The discharge rates at 8 days, 15 days, 22 days, and more than 22 days were 0.6%, 10.7%, 15.5%, and 73.2%, respectively. On day 22 of admission, the mean total BPRS score, positive scale, negative scale, and general scale were 30.4 ± 10.8, 13.2 ± 5.3, 7.5 ± 2.8, and 9.7 ± 3.2, respectively (Fig. 2). Total BPRS score at 8, 15, and 22 days was statistically significant decreased from the first day of admission (p-value < 0.001, < 0.001, and < 0.001, respectively).

Concerning the individual items of hallucination, grandiosity, hostile, and disorganized thought, the scores at admission date were 3.6 ± 0.9, 2.8 ± 1.1, 3.6 ± 0.9, and 3.2 ± 0.9, respectively. On day 22, the scores of these domains decreased by 1.8 ± 1.1, 1.2 ± 1.1, 1.8 ± 1.2, and 1.5 ± 1.1, respectively. Meanwhile, the individual items of negative scale which were emotional withdrawal, motor retardation, uncooperativeness, and blunted affect decreased from 3.0 ± 0.9, 2.4 ± 0.9, 3.3 ± 1.0, and 2.6 ± 0.9 to 1.8 ± 0.8, 1.3 ± 0.6, 1.6 ± 0.7, and 1.5 ± 0.6 at admission and day 22, respectively. In addition, the score of individual items on the general scale which were somatic concern, guilt feeling, and depressed mood also improved at day 22 when compared to the admission date. By day 22, anxiety and tension had improved slightly compared to the first day, with scores decreasing from 3.6 ± 0.8 and 3.6 ± 0.8 to 2.5 ± 0.7 and 2.1 ± 0.9, respectively.

The frequency of extrapyramidal symptoms (EPS) in our study was 4.4%, which were dysarthria (1.9%), acute dystonia (0.9%), akathisia (0.6%), mild cogwheel rigidity (0.6%), and bradykinesia (0.3%). Approximately 90.5% (287/317) of patients received oral trihexyphenidyl along with antipsychotic treatment at a mean dose of 5.5 ± 3.3 mg per day.

Linear regression analysis showed that there was no association between factors (sex, age, level of THC, duration of used cannabis, and duration of admission) and the change in BPRS score on day 22 compared to day 1 in all subscales.

With a focus on the chronic use of cannabis-induced schizophrenia, we found that 7% (21/317) of these patients had been later diagnosed with schizophrenia within the study period. There were 18 of 21 cases (86%) that used only cannabis. The rest of them used cannabis in combination with tramadol or benzodiazepines in which these two drug combinations did not induced psychosis. The median time to develop schizophrenia after discharge was 1.3 years (range 0.2-2.0 years). Of these, the average age of patients who develop schizophrenia was 20.9 ± 4.9 years old, with the first use of cannabis at 15.2 ± 2.1 years old. About 76.2% were unemployed and graduated from secondary or high school. Most of them used cannabis about 4.0 hole per day, and the duration of their used cannabis was 4 years. In addition, we found that 80% of our patients develop chronic cannabis use disorders (CUD) which usually presented with an inability to stop using cannabis, cravings, need to use higher amount, and impaired their social abilities.

## Discussion

The objective of this study was to determine the efficacy and safety of treatment CIP in patients who were diagnosed with CIP and had positive urine screening for THC at admission. Therefore, we can assure that our patients had psychosis related to the use of cannabis at admission. THC, a psychoactive substance, was commonly found in high amounts in the flowers of cannabis. *Cannabis sativa* which is the common species grown in Thailand [11]. THC elicits its acute psychoactive effect through the agonistic effect at the CB_1_ receptors [12]. However, there were some patients used other substances in combination with cannabis. Some of them (6.9%) had a positive urine test for methamphetamine. A combination of methamphetamine and cannabis may have a synergistic effect on psychosis by increasing dopamine level in the nucleus accumbens shell region [13]. Notably, the mean BPRS score for patients testing positive for both substances (58.6 ± 9.8) had a trend to have a higher mean BPRS score than those in THC-only positive patients (54.8 ± 9.6).

The legal status and perception of cannabis in Thailand have changed due to the withdrawal of dried leaves and flowers of cannabis plants, cannabis extracts and byproducts from extracting process with THC content no greater than 0.2% from the narcotic drugs list in 2020 [14]. The rate of CIP in our study, which was in the period between October 2013 and September 2019 (before delisting cannabis from narcotic drugs), was approximately 5% of total admission at PMNIDAT each year. However, data from the PMNIDAT website during 2019–2023 demonstrated that the prevalence of cannabis-related admission was rising drastically after cannabis legalization, especially in 2022 and 2023 (4.62%, 4.17%, 6.57%, 10.7%, and 16.31% in 2019 to 2023, respectively) [15].

Moreover, the demographic data of our study demonstrated that the average age of patients with CIP was in the range of 19–24 years old, and some of our patients were students (67/317; 21.1%). A comparison with two retrospective studies revealed a similar trend with the majority of patients were male and the average age were in the range of 21.0 years and 28.10 years, respectively [16, 17]. Notably, we found that patient below the age of eighteen accounted for approximately 10% (32/317) of those experienced psychosis from cannabis. The mean age of first cannabis use was 16.3 ± 3.4 years old and the lowest age in our study was 9 years old. Teenagers are more likely to try cannabis due to curiosity and a lack of knowledge regarding its long-term risks, such as cognitive impairment, and mood disorder. The studies on teenagers using cannabis indicated that THC has a more pronounced impact on cognitive decline in adolescents compared to adults [18, 19]. In addition, cannabis use in early adolescence carries a heightened risk of developing psychotic illnesses [20, 21]. THC-induced cognitive decline and poor judgement, which are usually not reversible, are related to effect of THC on CB1 receptors which are widely distributed throughout the central nervous system, especially enriched in the prefrontal cortex and the hippocampus. Thus, any disruption of the prefrontal-hippocampal pathway will negatively impact cognitive functions such as working memory, decision-making and inhibitory control [22]. These consequences highlighted the cautions of making cannabis easily accessible to general population, especially those who are under 18 years old.

For the treatment of CIP, nearly all of the patients received antipsychotics to target symptoms of CIP, especially hallucinations, delusions, irritability, and aggression. Antipsychotics play a crucial role by blocking D_2_ receptors in the mesolimbic tract, thereby mitigating dopamine-mediated signaling, which is the underlying cause of these symptoms. Risperidone and haloperidol were the most commonly used antipsychotics during acute and maintenance phase treatment. The median doses of risperidone and haloperidol used in this study were 2–4 mg/day and 5–20 mg/day, respectively. These dose range can effectively block D_2_ receptor. Thus, we could observe that the BPRS on day 8 following the administration of these antipsychotics showed a significant decrease of approximately 28% from the first day and subsequently decreased afterwards. However, the average risperidone equivalent dose of all antipsychotics during the maintenance phase in this study was 8.0 ± 5.9 mg/day, which was slightly higher compared to doses used in previous studies (6 mg/day) [8] and those used for treatment of first-episode psychosis (risperidone 3.3 mg/day) [23].

Risperidone was the most commonly prescribed medication both during the acute phase (83.6%) and the maintenance phase (85.5%). This was in accord with reviews and studies recommending the preference for atypical antipsychotics over typical antipsychotics in CIP for several reasons. Firstly, atypical antipsychotics demonstrate comparable efficacy to typical antipsychotics but with fewer adverse effects. Two randomized controlled trials found no significant difference in efficacy between typical and atypical antipsychotics, but atypical antipsychotics had a lower rate of extrapyramidal side effects [7, 8]. Secondly, atypical antipsychotics had beneficial effect on long-term cannabis use induced amotivational syndrome [24]. From our study, it was shown that risperidone could significantly improve all symptoms in negative domains of BPRS at day 22 when compared to baseline.

In our study, LAI antipsychotics were used more frequently in the maintenance phase (14.4%) compared to the acute phase (5.4%). Haloperidol decanoate was the most commonly used. However, LAI atypical antipsychotics such as paliperidone palmitate, and aripiprazole monohydrate, should be considered for treatment of CIP during maintenance phase. These drugs might control both positive and negative symptoms of CIP better than LAI conventional antipsychotics. Referring to 6.4% of our patients who were readmitted for CIP, LAI antipsychotics should be chosen which may help to improve adherence and prevent relapse in these CIP patients [25].

In addition to psychosis, high doses of cannabis usage led to the sudden onset of mood symptoms such as depression, fear, irrational panic, anxiety, and insomnia [26, 27]. Therefore, antidepressants and benzodiazepines were prescribed for mood symptoms in these patients. However, the anxiety and tension scores were still persisted at day 22. These may be related to cannabis withdrawal or the reduction of benzodiazepines dose in the maintenance phase.

Following the patients with CIP, we found that approximately 7.0% of patients (21 of 317) developed schizophrenia. A systematic review and meta-analysis by Murrie et al. in 2020 found that cannabis had the highest rate of developing schizophrenia compared to other substances (6 studies and 3040 people, 34%, 95% confidence interval 25–46%) [28]. It is confirmed that CIP could be an important risk factor for developing schizophrenia. In our study, this prevalence of cannabis-related primary psychosis (cannabis-induced schizophrenia) appeared to be lower than previous reports [28]. It might be due to our study design which was retrospective cross-sectional study conducted at a single institution. All data were collected solely from medical charts over a six-year period. Consequently, some cases of cannabis-related primary psychosis may have been missed due to lack of follow-up at our institution. However, the prevalence of schizophrenia resulting from cannabis use in Thailand may be rising, especially after the cannabis was removed from the narcotic drugs list, making it more easily accessible. The underlying mechanism of cannabis-induced schizophrenia may be explained by the impact of THC on CB1 receptors which are widely distributed in the sensory cortical circuits. Stimulation of CB1 receptor promotes neurogenesis and oligodendrogenesis in the central nervous system [29]. However, chronic THC use leads to downregulation of the CB1 receptors, potentially affecting these trophic and repair processes, ultimately contributing to irreversible brain damage associated with schizophrenia [30]. Furthermore, THC may induce a disproportionate downregulation of N-methyl-D-aspartate (NMDA) receptor, leading to hypofunction and implicating in schizophrenia [31]. The binding of THC at CB1 receptors also affect the myelination and synaptic pruning process of prefrontal cortex which is one of the last brain regions to develop during late adolescence. This area is responsible for many executive functions, including problem solving, working memory, abstract thinking, and increased inhibitory control [32]. Additionally, once patients with CIP who are in their teens develop schizophrenia, they can experience permanent cognitive impairment. There will be a tremendous impact in terms of manpower and economic burden. Thus, there is a pressing need to reevaluate the legalization of cannabis, given the observed of CIP or cannabis used-induced schizophrenia in our study. This phenomenon could lead to the potential loss of manpower, an increased burden on the healthcare system, and pose a hazard to families and others due to psychotic symptoms.

Our study had several limitations. First, the data were sourced from medical charts, potentially leading to some missing information. Secondly, changes in the BPRS scores may have been affected by the use of other psychotropic drugs. We were unable to control medication during the study because it was a retrospective observational study in real word data. Lastly, the lack of cognitive function recorded in medical charts may not adequately reflect the cognitive decline in individuals with CUD, which was found to be as high as 80%. In addition, the data from our study may not be extrapolated to other settings which have different context. Further prospective studies investigating the consequences of cannabis use and prevalence of cognitive impairment, and poor judgment, including impact of pharmacological treatment on these symptoms, should be conducted.

## Conclusions

Our study demonstrated that antipsychotics remained the primary pharmacological treatment option for both the acute and maintenance phases of CIP. They were commonly prescribed alone or in combination with benzodiazepines or antidepressants. Antipsychotics have shown efficacy in reducing the severity of psychosis symptoms, such as hallucination, delusion, grandiosity, and irritability, as well as addressing negative symptoms, typically within the first week of treatment. The average dose of antipsychotics, presented as risperidone equivalent dose, was found to be equal to or slightly higher than those used for the treatment of schizophrenia. Additionally, our study revealed that some CIP patients may develop schizophrenia even after successful treatment.

## Data Availability

Data is provided within the manuscript.

## Abbreviations

BPRS: Brief Psychotic Rating Scale
CB_1_: Cannabinoid receptor 1
CIP: Cannabis-induced psychosis
ICD-10: International Classification of Disease-10
IM: Intramuscular
ITT: Intention-to-treat
IV: Intravenous
LAI: Long-acting antipsychotics
PMNIDAT: Princess Mother National Institute on Drug Abuse Treatment
SPSS: Statistical Package for the Social Sciences
THC: Tetrahydrocannabinol
